# Identification and Comparison of *Colletotrichum* Secreted Effector Candidates Reveal Two Independent Lineages Pathogenic to Soybean

**DOI:** 10.3390/pathogens10111520

**Published:** 2021-11-21

**Authors:** Thaís R. Boufleur, Nelson S. Massola Júnior, Ísis Tikami, Serenella A. Sukno, Michael R. Thon, Riccardo Baroncelli

**Affiliations:** 1 Luiz de Queiroz College of Agriculture (ESALQ), University of São Paulo (USP), Piracicaba 13418-900, São Paulo, Brazil; nmassola@usp.br (N.S.M.J.); i.tikami@usp.br (Í.T.); 2 Department of Microbiology and Genetics, Institute for Agribiotechnology Research (CIALE), University of Salamanca, 37185 Villamayor, Salamanca, Spain; ssukno@usal.es (S.A.S.); mthon@usal.es (M.R.T.); 3 Department of Agricultural and Food Sciences (DISTAL), University of Bologna, Viale Fanin 44, 40126 Bologna, Italy

**Keywords:** anthracnose, genome sequencing, pathogenicity factors, *Colletotrichum truncatum*, *Colletotrichum orchidearum*, *Glomerella*, *Glycine max*

## Abstract

*Colletotrichum* is one of the most important plant pathogenic genus of fungi due to its scientific and economic impact. A wide range of hosts can be infected by *Colletotrichum* spp., which causes losses in crops of major importance worldwide, such as soybean. Soybean anthracnose is mainly caused by *C. truncatum*, but other species have been identified at an increasing rate during the last decade, becoming one of the most important limiting factors to soybean production in several regions. To gain a better understanding of the evolutionary origin of soybean anthracnose, we compared the repertoire of effector candidates of four *Colletotrichum* species pathogenic to soybean and eight species not pathogenic. Our results show that the four species infecting soybean belong to two lineages and do not share any effector candidates. These results strongly suggest that two *Colletotrichum* lineages have acquired the capability to infect soybean independently. This study also provides, for each lineage, a set of candidate effectors encoding genes that may have important roles in pathogenicity towards soybean offering a new resource useful for further research on soybean anthracnose management.

## 1. Introduction

Due to its agricultural versatility and nutritional content soybean (*Glycine max*) is the most produced legume worldwide; however, soybean yield is limited by anthracnose, a seed-borne disease caused by *Colletotrichum* spp. [1,2,3]. *Colletotrichum* is considered to be the eighth most important group of plant-pathogenic fungi due to its scientific and economic impact [4]. Currently, 257 species of *Colletotrichum* are accepted and classified into singletons (ST) or species complexes (s.c.) [5].

Since its first report in 1917 [6], *C. truncatum* has been considered the main species associated with soybean anthracnose, thus, the morphology, life cycle, variability and distribution of the species has been extensively investigated [7,8,9,10,11,12,13,14,15]. In the past five years, different species associated with the disease were reported, including *C. sojae*, *C. plurivorum* and *C. musicola*, that belong to the *C. orchidearum* s.c. [16,17,18,19]. A recent review revealed that several *Colletotrichum* species, belonging to nine s.c. and one ST, have been associated with soybean worldwide and that the *C. orchidearum* and the *C. truncatum* s.c. are the most common on symptomatic plants [2].

The evolutionary battle between plants and pathogens is marked by a dispute for survival and adaptation [20,21,22]. This arms race can be partially described by the “zig-zag” model [20], in which a pattern triggered immune response (PTI) is activated after the recognition of pathogen/damage-associated molecular patterns (PAMPs/DAMPs) [20,23,24]. On the other hand, pathogens can bypass this layer of defense by secreting effectors, defined as proteins that interfere in the structure or processes of the host cell, reducing the defense responses and/or improving access to nutrients, allowing the colonization of the host by the pathogen [23]. When pathogen effectors and/or effector targets are detected by the host’s resistance (*R*) genes the second layer of defense, called effector-triggered immunity (ETI) is activated and can result in a hypersensitive reaction (HR) [21,23]. With the advance of molecular studies, it was shown that the division among PTI and ETI is blurred [24,25,26], and now an integrated plant immune system has been proposed, where a crosstalk between plant immune receptors is essential to both, PTI and ETI achieve its maximum immune response [27].

The increasing need to feed the growing population demands a maximization of production, that is mostly achieved by monoculture. The consequence of this strategy is the homogenization of agricultural environments, that can influence the co-evolutionary arms race among plants and pathogens providing selective advantages to pathogens [28]. In this scenario, the knowledge of the mechanisms involved in the pathogenicity and, the understanding of the differences between different *Colletotrichum* species is a key step for the development and improvement of soybean anthracnose control strategies.

With the rapid expansions of sequencing technologies and computational tools, the analyses and comparison of whole genomes has become a common practice [29], allowing the establishment of cause and effect correlations among genome features and the biology of plant pathogens [30,31]. To date, 43 genomes of *Colletotrichum* have been released [32,33,34,35,36,37,38,39,40,41,42,43,44,45,46,47,48,49,50,51,52,53,54,55,56] (http://www.colletotrichum.org/genomics/, accessed on 15 November 2012), including the genomes of the soybean pathogenic species *C. truncatum*, *C. musicola*, *C. plurivorum* and *C. sojae* [54].

It is known that the evolution through adaptation of pathogens to different hosts can involve sets of effectors, that can specialize to infect a specific host [57,58,59,60,61], therefore the evolutionary trajectory of host-pathogen interactions can help to clarify the mechanisms underlying the threat of pathogens to crops [62]. The identification of effector candidates is the first step into the functional characterization of these molecules. Until now, several studies on effectors of different species of *Colletotrichum* such as *C. higginsianum* [63,64] *C. orbiculare* [65,66] *C. lentis* [67,68,69], *C. graminicola* [70,71,72] *C. simmondsii*, *C. fiorinae*, *C. nymphaeae*, *C. salicis* [39], *C. lindemunthianum* [73], *C. falcatum* [74], *C. fruticola*, *C. siamense*, *C. aenigma*, *C. tropicale*, *C. viniferum* [53] have been published. On the other hand, comparative genomic studies of *Colletotrichum* spp. that infect soybean have not been performed and the number of candidate effectors of *C. truncatum*, *C. plurivorum*, *C. musicola* and *C. sojae*, and how many are unique to these species is unknown. A compilation of candidate effectors of those species may help to identify determinants of host specificity in the *Colletotrichum*-soybean interaction as well as better understanding the mechanisms underlying soybean infection.

To gain a better understanding of the evolutionary origin of soybean anthracnose, we analyzed the repertoire of Lineage Specific Effector Candidates (LSECs) defined as secreted proteins that have no homology to any other protein or that have homology to proteins from other members of the same genus, species or s.c. [39]. We analyzed the proteomes encoded by 12 *Colletotrichum* species: four pathogenic to soybean (*C. truncatum*, *C. musicola*, *C. plurivorum* and *C. sojae*), and eight non-pathogenic to this host. To check the intraspecific variability of the identified *C. truncatum*-LSECs, whole genome data of 18 *C. truncatum* strains isolated from soybean were assembled and scanned [14]. Moreover, to support our results, RNA-sequencing (RNAseq) data were used to confirm the expression of *C. truncatum*-LSECs. This work provides a useful platform for future functional studies aimed to clarify the role of *Colletotrichum* spp. LSECs in soybean anthracnose and shed light, for the first time on the genetic mechanisms of *Colletotrichum* spp. specialization to soybean.

## 2. Results

### 2.1. Among the Selected Colletotrichum Species, Only C. truncatum and Members of the C. orchidearum s.c. Are Pathogenic to Soybean

The pathogenicity of 10 *Colletotrichum* species selected for comparative genomic analyzes (Table 1) were tested on soybean.

**Figure 1:**
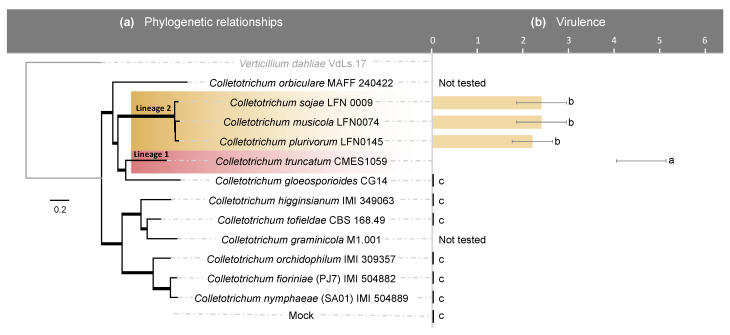
Evolutionary relationships of *Colletotrichum* species. (**a**) Bayesian inference phylogenetic of the strains used in this study. The tree was reconstructed from concatenated nucleotide alignments of the *ACT* (*actin*), CHS-1 (*chitin synthase*), and *GAPDH* (*glyceraldehyde 3-phosphate dehydrogenase*) genes. For each locus the alignment was performed with MAFFT v7.450 [76], exported to MEGA7 [77] and the best-fit substitution model was calculated. Thicker branches represent nodes with Bayesian posterior probability equal to 1.00. The scale bar represents the number of expected substitutions per site. (**b**) Level of virulence of *Colletotrichum* species to soybean. Tukey’s test was applied on transformed data ((X + 1)ˆ0.5). Equal letters do not differ in the average of virulence among *Colletotrichum* strains in the Tukey test with *p*-value = 0.05%. Species belonging to the *C. orchidearum* species complex (s.c.) are represented by yellow bars, while *C. truncatum* is represented by the pink bar.

Assays confirmed that only *C. truncatum* and the three species belonging to the *C. orchidearum* s.c., *C. musicola*, *C. plurivorum* and *C. sojae* cause anthracnose symptoms in soybean, of which *C. truncatum* is the most virulent to the tested soybean cultivar (Monsoy IPRO7739) than the three species belonging to the *C. orchidearum* s.c. (Figure 1). *Colletotrichum gloeosporioides*, *C. higginsianum*, *C. tofieldiae*, *C. orchidophilum*, *C. fioriniae* and *C. nymphaeae* were not pathogenic to soybean (Figure 1).

### 2.2. The Majority of Candidate Effectors of Colletotrichum Species Are Conserved

To better understand the evolutionary aspects of the two main *Colletotrichum* s. c. that infect soybean worldwide (*C. truncatum* s.c. and *C. orchidearum* s.c.) [2] we conducted in silico analyzes (Figure S1) to check if the representative species of those complexes (*C. truncatum*, *C. musicola*, *C. plurivorum* and *C. sojae*) share a unique set of effector candidates.

**Figure 2:**
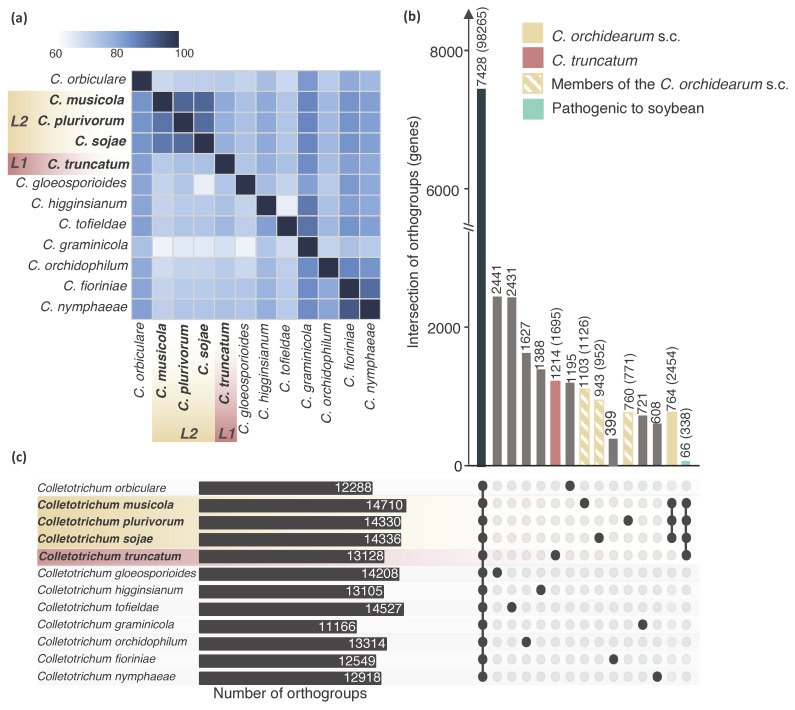
Comparative genomic analysis of *Colletotrichum* species pathogenic and not pathogenic to soybean. Species highlighted in yellow represent the *C. orchidearum* species complex (s.c.), and striped yellow bars correspond to each species belonging to the *C. orchidearum* s.c. (*C. musicola*, *C. plurivorum* and *C. sojae*); while *C. truncatum* is represented in red. (**a**) Heatmap showing the percentage of overlapping proteins shared in pairwise comparisons (values correspond to percentage of proteins encoded by the species reported in the y axis that show similarity with those reported by the species reported in the *x* axis). (**b**) UpsetR plot of the protein clustering analysis of 12 *Colletotrichum* species. Bars on the upper side represent the number of orthogroups shared by the species highlighted by the black dots reported on the bottom side. The number of genes corresponding to the orthogroups is in parentheses. (**c**) Species compared in this study, the bars on the right side of the species name represent the total number of orthogroups in each proteome. L1 (lineage 1); L2 (lineage 2).

The proteomes of the 12 *Colletotrichum* species (Table 1) were assigned to 32,018 orthogroups, of which 7428 are shared among all the proteomes analyzed (Figure 2B). Comparative analysis identified 66 orthogroups comprising 338 genes of *Colletotrichum* spp. common only to the four species infecting soybean, of which only one orthogroup is fully secreted; and 764 orthogroups (2454 genes) shared only between the species belonging to the *C. orchidearum* s.c., of which eight orthogroups are secreted. While 1214 (1695 genes); 1103 (1126 genes); 760 (771 genes) and 943 (952 genes) orthogroups were specific to *C. truncatum*, *C. musicola*, *C. plurivorum* and *C. sojae*, respectively (Figure 2C).

The proteomes of the four soybean infecting species of *Colletotrichum* were scanned for the presence of signal peptides, transmembrane (TM)-domains, and glycosylphosphatidylinositol (GPI)-anchors. For further analyses, the secretome of each *Colletotrichum* species was defined based on those proteins with a predicted signal peptide, and absence of TM domains [78] and GPI-anchors. The secretomes of the four species vary between 9–10%, being 1638; 1485; 1495; and 1447 proteins for *C. truncatum*, *C. musicola*, *C. plurivorum* and *C. sojae* respectively (Table 2).

**Figure 3:**
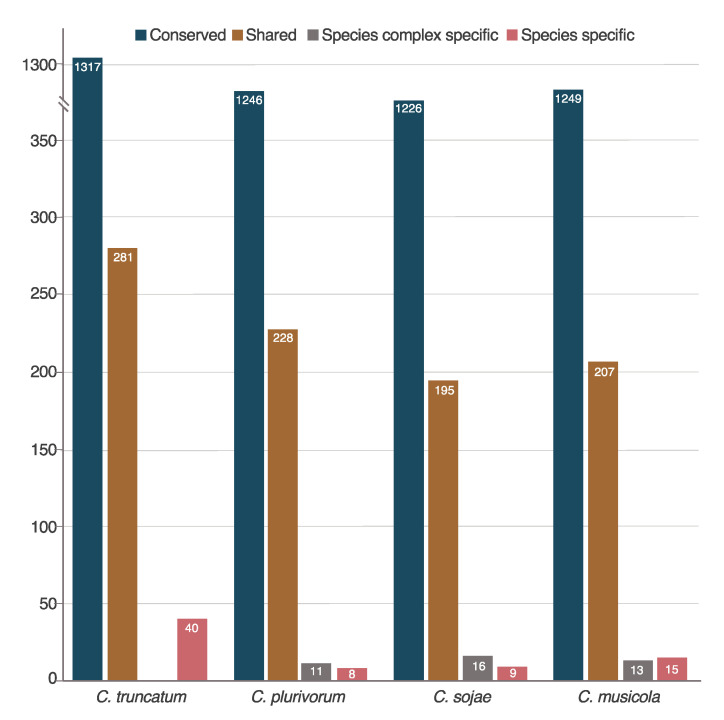
Effector candidates of *Colletotrichum* species pathogenic to soybean. Effector candidates of *C. musicola*, *C. plurivorum*, *C. sojae* and *C. truncatum* with similarity outside the genus *Colletotrichum* are represented in dark blue, while effector candidates with similarity with other species of the genus are represented in light brown. *C. orchidearum*-Lineage Specific Effector Candidates (LSECs) are represented in gray and species-LSECs are represented in light red. Total numbers of candidate effectors are represented in the bars.

Our results revealed that most of the effector candidates of the four *Colletotrichum* species pathogenic to soybean are present in other microorganisms, corresponding to 80% of *C. truncatum*, 84% of *C. musicola*, 83%, of *C. plurivorum* and 85% of *C. sojae*. While around 15% of the effector candidates of each species are shared only among the *Colletotrichum* genus (Figure 3). LSECs, with no similarity inside or outside the genus *Colletotrichum* were identified, among those, 11 *C. orchidearum*-LSECs in *C. plurivorum*, 13 *C. orchidearum*-LSECs in *C. musicola*; and 16 *C. orchidearum*-LSECs in *C. sojae*. We also identified 40 *C. truncatum*-LSECs, 15 *C. musicola*-LSECs, eight *C. plurivorum*-LSECs and nine *C. sojae*-LSECs. Host-LSECs shared only between the four *Colletotrichum* species that infect soybean were not identified (Figure 3, Table S1). The corresponding orthogroups of all the sets of s.c. and species-LSECs aforementioned were assigned to their corresponding orthogroups based on the similarity analysis of the proteins (Table S1).

The absence of similarity to proteins with a known function is a common characteristic to effector proteins [78]. All the LSECs were scanned with RunIprScan (http://michaelrthon.com/runiprscan/, accessed on 15 February 2021) to identify conserved domains and submitted to a BLAST against the non-redundant database Pathogen Host Interactions-base (PHI-base) to check the similarity with known genes of other microorganism species. All LSECs of the four *Colletotrichum* spp. pathogenic to soybean do not have any known domain or similarity in PHI-base (Table S1).

We scanned the *C. orchidearum* s.c. and species-LSECs for characteristics commonly observed in effector proteins, such as a high percentage of cysteines (cysteine-rich), with >2% of cysteines in their amino acid sequences [79], repeat-containing proteins [80] and the predicted translocation to different subcellular compartments of the plant cell, such as the chloroplast or mitochondria when they have a transit peptide, to the plant cell nucleus, when they possess nuclear localization signals (NLS) [81] or are delivered to the plant apoplast [82]. All *C. orchidearum*-LSECs, have at least one of the above-mentioned characteristics, from those, six; five; and five LSECs were predicted as effectors by EffectorP 2.0 tool for *C. musicola*, *C. plurivorum*, and *C. sojae*. Among the species-LSECs, 11 *C. musicola*, seven of *C. plurivorum* and eight of *C. sojae* have at least one of these characteristics, of which five, two and three were predicted to be effectors by EffectorP 2.0 tool. Among the *C. truncatum*-LSECs, 34 were predicted to have at least one of those characteristics, being 16 of them predicted by EffectorP 2.0 (Table 3 and Table S1).

### 2.3. C. truncatum LSECs Are Expressed and Have Evolutionary Evidence

**Figure 4:**
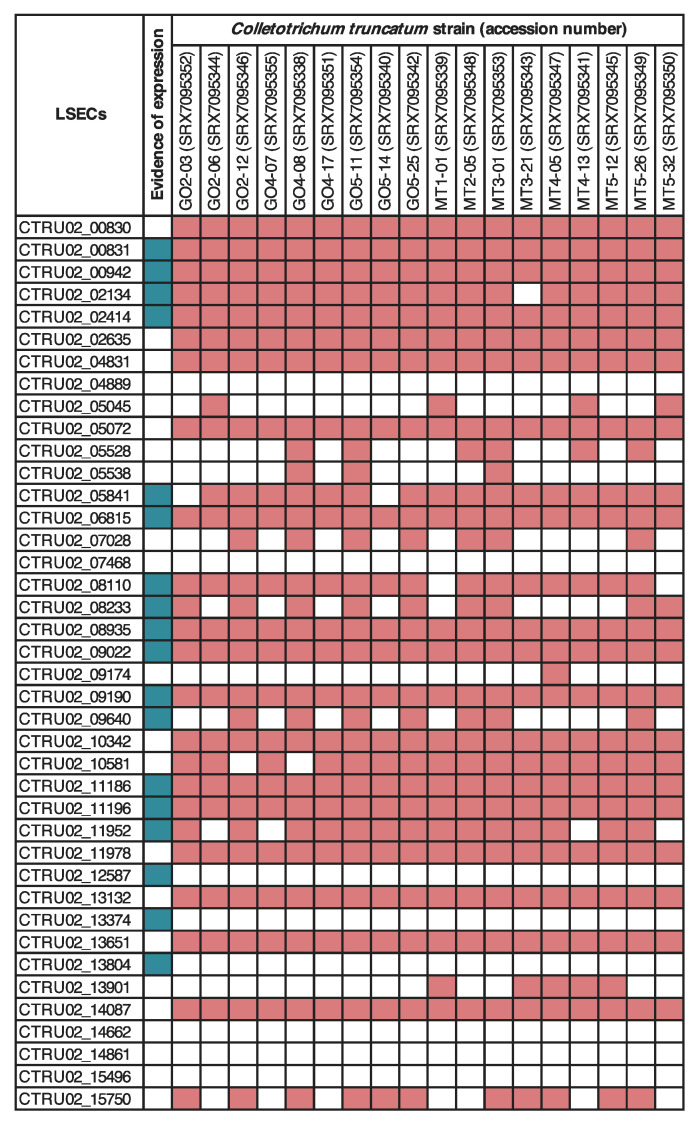
The distribution of 58 LSECs in 18 strains of *Colletotrichum truncatum* and evidence of expression of *C. truncatum* (CMES1059). 18 genomes of *C. truncatum* pathogenic to soybean were scanned for presence/absence of the 58 *C. truncatum*-LSECs using BLAST. Red squares indicate the presence of LSECs by the blasting with coverage >90% and identity >60%. Green squares indicate evidence of expression of LSECs in soybean and/or in vitro.

To confirm the expression of *C. truncatum*-LSECs in vitro and in soybean during the infection by *C. truncatum*, samples were collected for RNA sequencing at 12;48 and 120 hpi, and 21 cDNA libraries were sequenced. A total of 1,202,535,286 raw reads were generated by Illumina HiSeq4000 sequencing. Overall, from 0.02 to 7.56% of the paired-end reads were mapped to the *C. truncatum* genome. 18 *C. truncatum* LSECs have evidence of expression *in planta* and/or in vitro. From those, nine are evolutionarily conserved in 18 *C. truncatum* genomes pathogenic to soybean. Another eight *C. truncatum* LSECs are conserved but are not expressed (Figure 4).

## 3. Discussion

The availability of four representative *Colletotrichum* genomes of the *C. truncatum* s.c. and *C. orchidearum* s.c. [54] reported as the most distributed s.c. associated with soybean worldwide [2], along with the genomes of several *Colletotrichum* species associated with other hosts [32,33,34,35,36,37,38,39,40,41,42,43,44,45,46,47,48,49,50,51,52,53,54,55], allowed us to investigate the evolutionary origin of soybean anthracnose, by looking at the repertoire of effector candidates of each species and comparing them with the proteomes of eight additional *Colletotrichum* species non-pathogenic to soybean.

Effectors proteins produced by plant pathogens are secreted proteins, many of which translocated to the apoplast or cytoplasm of the host, where they alter the host defense responses to allow colonization by the pathogen [23,83]. Prediction of effector proteins from proteomes of *Colletotrichum* species has revealed different sets of effector candidates [42,56,69,75]. The evolution of effector proteins rely on the arms-race between plants and pathogens, with the aim of escape detection and evolve the capability of cause disease in different hosts [30], therefore the pathogenicity to specific hosts and/or cultivars can be a result of the evolution of effector proteins from a common ancestor [84,85], as shown for the hemibiotrophic pathogen *Phythophthora infestans* [21], *Venturia* spp. [86] and *Ceratocystis* spp. [87]. Our results revealed effector candidates for the four species pathogenic to soybean. Most of the *C. orchidearum* s.c. and species-LSECs are predicted to be secreted to the plant apoplast, while only a few genes are predicted to be localized to the plant cell nucleus or other subcellular compartments (Table 3 and Table S1). These results suggest that the initial contact with the host is determinant for the capability of *Colletotrichum* species to infect soybean.

Initial pathogenicity tests revealed that among the tested *Colletotrichum* isolates, only the four *Colletotrichum* species previously associated with soybean [6,16,17,18] were pathogenic to the evaluated soybean cultivar. The three species that belong to the *C. orchidearum* s.c. showed a similar level of virulence, and lower than the level of virulence of *C. truncatum.* In another study, the virulence of one isolate of *C. plurivorum* was compared with five isolates of *C. truncatum*, and overall, the isolate was less virulent than at least one isolate of *C. truncatum* in soybean pods, stems and cotyledons, moreover, the authors reported that pod twisting symptoms were only caused by *C. plurivorum*, when the same stage of soybean development was compared after inoculation with *C. truncatum* [88].

While *C. truncatum* has been associated with soybean since 1917 [6], *C. musicola*, *C. plurivorum* and *C. sojae* were detected in soybean fields only recently [16,17,18]. Studies have revealed that the *C. orchidearum* s.c. has been misidentified at least since 2003, being *C. truncatum* and *C. orchidearum* s.c. the most associated with soybean until now [2]. Our results show that the four species that infect soybean belong to two lineages and do not share any of the identified LSECs. Moreover, the estimated divergence time of the *C. truncatum* s.c. occurred around 22.9 million years ago (mya), while the *C. orchidearum* s.c diverged 4.8 mya [89], both of them before the domestication of soybean, that occurred 3000 years ago in China [90]. This evolutionary evidence, along with experimental data and the absence of host-LSECs shared only among the four species of *Colletotrichum* that infect soybean, strongly suggests that the two main *Colletotrichum* lineages associated with soybean have acquired the capability to infect soybean independently. Currently, *C. truncatum* is the most important species associated with soybean anthracnose worldwide [8,91], therefore, we checked if the *C. truncatum*-LSECs are conserved in 18 additional *C. truncatum* genomes pathogenic to soybean. Our results revealed that 17 *C. truncatum* genes have evolutionary evidence of being conserved among the species. This suggests that those effectors might play a role in the virulence of *C. truncatum* to soybean, as microorganisms do not keep useless genes due the high fitness costs of maintaining effector alleles [62,92]. Additionally to in silico prediction based genome sequences, an initial list of effector candidates can be narrowed down based on their expression [78]. 18 *C. truncatum*-LSECs have evidence of expression in soybean and/or in vitro. The low coverage of RNAseq data was a limiting factor for the analysis of gene expression, therefore LSECs without evidence of expression should not be excluded from the initial dataset and be further investigated.

The identification of sets of LSECs of the *C. orchidearum* s.c. and *C. truncatum* open the field to perform evaluations of the functional role of these genes in soybean infection. Besides cultural and chemical control strategies that have already been described for soybean anthracnose, recent outbreaks of the disease have been reported by researchers and producers [8,14,88], suggesting that the control strategies used are not always effective. This may be a consequence of different *Colletotrichum* species present in soybean fields, that allied to the suggestion of separate evolution of these species, may imply directly in disease management strategies, as the correct identification of the causal agent is crucial to an efficient control strategy [93,94].

## 4. Conclusions

This work sheds light on the evolutionary aspects of *Colletotrichum* spp. associated with soybean anthracnose. Our results suggest that there are at least two distinct lineages that evolved the capability to infect soybean independently. These results are supported by the identification of different sets of LSECs in all the four species compared, and the absence of shared genes only among the four species that infect soybean. Moreover, the level of virulence of species of the *C. orchidearum* s.c. is lower when compared to *C. orchidearum*. We confirmed that 42% of *C. truncatum*-LSECs are conserved in 18 re-sequenced genomes, while 25% of those also have evidence of expression in planta and/or in vitro. The presence of isolate-SECs with evidence of in planta expression opens new perspectives linking these loci with virulence.

Recent outbreaks of the disease reported by researchers and producers [14,91,95] suggest that the cultural and chemical strategies that have been used to control soybean anthracnose are not always effective. This may be related to the different lineages of *Colletotrichum* present in soybean fields. Evolutionarily distinct lineages may require the application of multiple and specific disease management strategies.

A platform of LSECs of *C. truncatum*, *C. plurivorum*, *C. sojae* and *C. musicola* is now provided. These loci can be used for functional studies and, once their function has been confirmed, as targets for breeding programs.

## 5. Materials and Methods

### 5.1. Fungal Strains Used

Twelve *Colletotrichum* species were selected for comparative genomic analyses (Table 1). From those, four are pathogenic to soybean, including *C. musicola*, *C. plurivorum* and *C. sojae*, part of the *C. orchidearum* s.c. and *C. truncatum*; while the other eight, including: *C. orbiculare*, *C. gloeosporioides sensu lato*, *C. higginsianum*, *C. tofieldiae*, *C. graminicola*, *C. orchidophilum*, *C. fioriniae* and *C. nymphaeae*, are pathogenic to other hosts (Table 1).

### 5.2. Pathogenicity Assays

Pathogenicity assays were performed to confirm the capability of the selected *Colletotrichum* strains to cause soybean anthracnose (Table 1). For sporulation, strains were cultured on Potato Dextrose Agar (PDA) medium (Sigma-Aldrich, St. Louis, MO, USA) for 15 days at 25 °C and conidia suspensions were prepared and adjusted to 1 × 10^6^ conidia/mL.

Soybean seeds of the IPRO7739 cultivar (Monsoy company, São Paulo, Brazil), were superficially disinfected for 1 min into a 1% NaClO solution, rinsed three times in sterile distilled water (SDW) and placed in Petri dishes containing 100 g of sterile sand, soaked with 10 mL of SDW. Seeds were incubated in the dark for 32 h at 25 °C.

Conidia suspensions of each *Colletotrichum* strain were placed on five germinated seeds as described previously [96]. SDW was used as a negative control. Inoculated seedlings were incubated for 4 h, transferred to pots filled with vermiculite and transferred to a greenhouse for 7 days. The virulence of *Colletotrichum* strains was evaluated using an adapted diagrammatic scale that ranges from 0 to 5 [15]. Severity data were analyzed with the post-hoc Tukey method at 0.05 significance level, using the ExpDes R package (v.1.2.0) (Alfenas, Brazil).

### 5.3. Identification of Specific Effector Protein Candidates (SECs) of Soybean Pathogenic Colletotrichum Species

The proteomes of four *Colletotrichum* species pathogenic to soybean, and eight non-pathogenic species were analyzed (Table 1). A phylogeny of the 12 selected proteomes was reconstructed based on the combined *actin* (*ACT*), *chitin synthase* (*CHS*) and *glyceraldehyde-3-phosphate dehydrogenase* (*GAPDH*). *Verticillium dahliae* (VdLs.17) was used as an outgroup. Random trees were sampled every 1000 generations, and the analyses were run for 5,000,000 generations using MrBayes (v. 3.2.7) (Oxford, England). The predicted proteomes of the 12 *Colletotrichum* spp. were clustered based on similarity with OrthoFinder (v.2.3.5) (Oxford, England) [95] and the clusters of proteins were visualized with the R package UpsetR (v.1.4.0) [97] to identify shared and specific orthogroups between the species and s.c.

To predict the set of effector candidates of *C. truncatum*, *C. musicola*, *C. plurivorum* and *C. sojae*, publicly available proteomes were used [54]. Proteins containing a signal peptide cleavage site were predicted with SignalP (v.5.0) (Lingby, Denmark) [98], then sequences containing TM-domains and GPI-anchors were identified using THMMM (v.2.0) (Lingby, Denmark) [99] and PredGPI (http://gpcr.biocomp.unibo.it/predgpi/, accessed on 20 February 2021) [100]. The initial set of effector candidates for each species of *Colletotrichum* included those proteins that are predicted to have a signal peptide cleavage site, no TM-domains and no GPI-anchors.

The set of effector candidates of each *Colletotrichum* species was submitted individually to a series of BLAST searches with an *e*-value cutoff of 1E-5 and classified into shared (proteins with homology to proteins from other members of the genus *Colletotrichum*), s.c. specific (those that had homology only within other species from the same s.c.), host-specific (shared only between the four species that infect soybean) and species-specific (those that had no homology to any other protein either within or outside of the same genus) LSECs [39]. The final set of predicted LSECs was scanned with RunIprScan (http://michaelrthon.com/runiprscan/, accessed on 15 February 2021) to identify conserved domains and submitted to a BLAST search against the non-redundant database (nr db) of NCBI and PHI-base to check the similarity with known genes of other microorganism species; being considered conserved those proteins with similarity outside the genus *Colletotrichum*.

Species-specific and species complex LSECs were characterized. For the prediction of subcellular localization within the plant cell, mature protein sequences were submitted to LOCALIZER (http://localizer.csiro.au/, accessed on 24 February 2021) [81], and to the prediction of apoplastic LSECs, the proteins were submitted to ApoplastP (http://apoplastp.csiro.au/, accessed on 24 February 2021) [82]. The percentage of cysteines was identified in Geneious (v.2020.10.4) (San Diego, CA, USA) and repeat-containing proteins were predicted using T-REKs [101].

### 5.4. Genome Assembly and Gene Evolution

To check if *C. truncatum*-LSECs are conserved among the species, Illumina reads of 18 *C. truncatum* strains available in NCBI (Table 4) were trimmed with Trim Galore (v.0.4.5) (Cambridge, UK) Forward and reverse reads were merged using Flash (v.1.2.7) (Baltimore, MD, USA) [102]. Assemblies of combined and uncombined reads were performed with SPAdes v.3.13.1 [103] (St. Peterspurg, Russia) using *the C. truncatum* CMES1059 strain genome as a reference.

### 5.5. Evidence of Expression of C. truncatum by RNAseq

To confirm evidence of gene expression of *C. truncatum* in planta, five pre-germinated seeds of soybean cultivars IPRO7739 and IPRO8372 (Monsoy company, São Paulo, Brazil) were inoculated with *C. truncatum* (CMES1059) strain as described in 5.2. Hypocotyls fragments of 0.5 cm of five randomly selected plants were collected and pooled together at 12; 48 and 120 hpi. To confirm evidence of expression of *C. truncatum* in vitro, 100 mL of potato dextrose liquid culture was inoculated with *C. truncatum* conidia in 250 mL Erlenmeyer flasks at 25 °C, shaken at 150 rpm. After 120 hpi micelia was collected by filtration and washed with SDW. Harvested plant tissue and fungal micelia was flash-frozen in liquid N_2_ and stored at −80 °C until RNA extraction. Three biological replicates of the experiment were performed. The collected material was ground using mortar and pestle and total RNA was purified using PureLink RNA Mini Kit (Invitrogen, Carlsbad, CA, USA) following the manufacturer-s instructions. Total RNA was treated with RNAse-free DNAse (Thermo Fisher Scientific, Waltham, MA, USA) to remove DNA contamination. The quantity of total RNA was estimated using Qubit 2.0 flurometer (Thermo Fisher Scientific, Waltham, MA, USA) and RNA integrity was checked using Agilent TapeStation 4200 (Agilent Technologies, Palo Alto, CA, USA).

Total extracted RNA was sent to Genewiz (South Plainfield, NJ, USA) for Illumina sequencing. In total, 21 libraries derived from all the treatments were prepared using NEBNext Ultra RNA Library Prep Kit for Illumina (NEB, Ipswich, MA, USA) using manufacturer’s instructions. Sequencing libraries were validated on the Agilent TapeStation (Agilent technologies, Palo Alto, CA, USA) and quantified in Qubit 2.0 fluorometer (Invitrogen, Carlsbad, CA, USA) and by quantitative PCR (Kapa Biosystems, Wilmington, NC, USA). Libraries were sequenced using Illumina HiSeq4000 (2 × 150 bp) (Illumina, San Diego, CA, USA).

The quality of reads was accessed using FastQC (v.0.11.7) (Cambridge, UK) and clean reads were obtained by removing reads containing adapters with CutAdaptors (v.1.9.1) (Uppsala, Sweden). Paired-end clean reads were mapped against the *C. truncatum* CMES1059 reference genome [54] using HISAT (v.2.1.0) (Baltimore, MD, USA). Alignments from each library were processed with StringTIE (v.1.3.5) (Baltimore, MD, USA) to quantify expression values of transcripts.

## Figures and Tables

**Table 1:** *Colletotrichum* strains used in the pathogenicity test and comparative genomics analysis.

Strain	Species	Species Complex	Host	Origin	*
MAFF 240422	*C. orbiculare*	*C. orbiculare*	*Cucumis sativus*	Japan	[47]
LFN0074	*C. musicola*	*C. orchidearum*	*Glycine max*	Brazil	[54]
LFN0145	*C. plurivorum*	*C. orchidearum*	*Glycine max*	Brazil	[54]
LFN0009	*C. sojae*	*C. orchidearum*	*Glycine max*	Brazil	[54]
CMES1059	*C. truncatum*	*C. truncatum*	*Glycine max*	Brazil	[54]
Cg-14	*C. gloeosporioides s.s.*	*C. gloeosporioides*	*Persea americana*	Israel	[34]
IMI 349063	*C. higginsianum*	*C. destructivum*	*Brassica rapa*	Trinidad & Tobago	[75]
CBS 168.49	*C. tofieldiae*	*C. spaethianum*	*Lupinus polyphyllus*	Germany	[41]
M1.001	*C. graminicola*	*C. graminicola*	*Zea mays*	USA	[32]
IMI 309357	*C. orchidophilum*	none	*Phalaenopsis* sp.	United Kingdom	[45]
IMI 504882	*C. fioriniae*	*C. acutatum*	*Fragaria x ananassa*	New Zealand	[29]
IMI 504889	*C. nymphaeae*	*C. acutatum*	*Fragaria x ananassa*	Denmark	[39]

* Reference of the genome sequences.

**Table 2:** Secretome size of the four species of *Colletotrichum* that infect soybean, compared in this study.

Species	Proteome	Signal Peptide	Absence of TM/GPI Anchor	% of Secreted Proteins
*C. truncatum*	15,901	2116	1638	10
*C. plurivorum*	15,153	1989	1495	10
*C. sojae*	16,124	1931	1447	9
*C. musicola*	16,826	1871	1485	9

**Table 3:** Predicted *C. orchidearum* s.c. and species-LSECs of the four species of *Colletotrichum* pathogenic to soybean, containing characteristics commonly associated with effector proteins in fungi, and the total number of protein sequences predicted as effectors by EffectorP 2.0.

*C. orchidearum* s.c. SECs							
Species	LSECs	RCP	SL (NLS)	SL (Other)	Apoplast	CR	EffectorP
*C. musicola*	13	5	0	0	9	7	6
*C. plurivorum*	11	5	1	0	7	6	5
*C. sojae*	16	3	2	2	11	8	5
**Species-LSECs**							
*C. truncatum*	40	7	5	2	16	21	16
*C. musicola*	15	2	0	2	4	8	5
*C. plurivorum*	8	0	0	4	1	4	2
*C. sojae*	9	2	0	1	2	6	3

LSECs: Lineage Specific Effector Candidates; RCP: repeat-containing proteins; SL: subcellular localization; NLS: nuclear localization signal; CR: cysteine-rich proteins (>2%); NA: not applicable.

**Table 4:** *Colletotrichum truncatum* strains used in the evolutionary analysis.

Strain	Species	Species Complex	Host	Origin	Accession N°
MT1-01	*C. truncatum*	*C. truncatum*	*Glycine max*	Brazil	SRX7095338
MT2-05	*C. truncatum*	*C. truncatum*	*Glycine max*	Brazil	SRX7095339
MT3-01	*C. truncatum*	*C. truncatum*	*Glycine max*	Brazil	SRX7095348
MT3-21	*C. truncatum*	*C. truncatum*	*Glycine max*	Brazil	SRX7095349
MT4-05	*C. truncatum*	*C. truncatum*	*Glycine max*	Brazil	SRX7095350
MT4-13	*C. truncatum*	*C. truncatum*	*Glycine max*	Brazil	SRX7095351
MT5-12	*C. truncatum*	*C. truncatum*	*Glycine max*	Brazil	SRX7095352
MT5-26	*C. truncatum*	*C. truncatum*	*Glycine max*	Brazil	SRX7095353
MT5-32	*C. truncatum*	*C. truncatum*	*Glycine max*	Brazil	SRX7095354
GO2-03	*C. truncatum*	*C. truncatum*	*Glycine max*	Brazil	SRX7095355
GO2-06	*C. truncatum*	*C. truncatum*	*Glycine max*	Brazil	SRX7095340
GO2-12	*C. truncatum*	*C. truncatum*	*Glycine max*	Brazil	SRX7095341
GO4-07	*C. truncatum*	*C. truncatum*	*Glycine max*	Brazil	SRX7095342
GO4-08	*C. truncatum*	*C. truncatum*	*Glycine max*	Brazil	SRX7095343
GO4-17	*C. truncatum*	*C. truncatum*	*Glycine max*	Brazil	SRX7095344
GO5-11	*C. truncatum*	*C. truncatum*	*Glycine max*	Brazil	SRX7095345
GO5-14	*C. truncatum*	*C. truncatum*	*Glycine max*	Brazil	SRX7095346
GO5-25	*C. truncatum*	*C. truncatum*	*Glycine max*	Brazil	SRX7095347

## Data Availability

All the data analyzed in this work are publicly available at NCBI (https://www.ncbi.nlm.nih.gov/ accessed on 15 November 2020) under the accession numbers: *C. orbiculare* (AMCV00000000.1), *C. musicola* (WIGM00000000), *C. plurivorum* (WIGO00000000), *C. sojae* (WIGN00000000), *C. truncatum* (VUJX00000000.1), *C. gloeosporioides* (AMYD00000000.1), *C. higginssianum* (LTAN00000000.1), *C. tofieldiae* (LFHQ00000000.1), *C. graminicola* (ACOD00000000.1), *C. orchidophilum* (MJBS00000000.1), *C. fiorineae* (JARH00000000.1), *C. nymphaeae* (JEMN00000000.1).

## Supplementary Materials

The following are available online at https://www.mdpi.com/article/10.3390/pathogens10111520/s1. Figure S1: In silico analysis workflow for the prediction of effector candidates in *C. truncatum*, *C. musicola*, *C. plurivorum* and *C. sojae.* Table S1: Lineage Specific Effector Candidates (LSECs) of *C. truncatum*, *C. musicola*, *C. plurivorum* and *C. sojae*.
