# Supplementary figures and images for: Identification and Comparison of Colletotrichum Secreted Effector Candidates Reveal Two Independent Lineages Pathogenic to Soybean
Source: Pathogens. 2021 Nov 21;10(11):1520. doi: 10.3390/pathogens10111520 (PMC8625359; doi:10.3390/pathogens10111520)

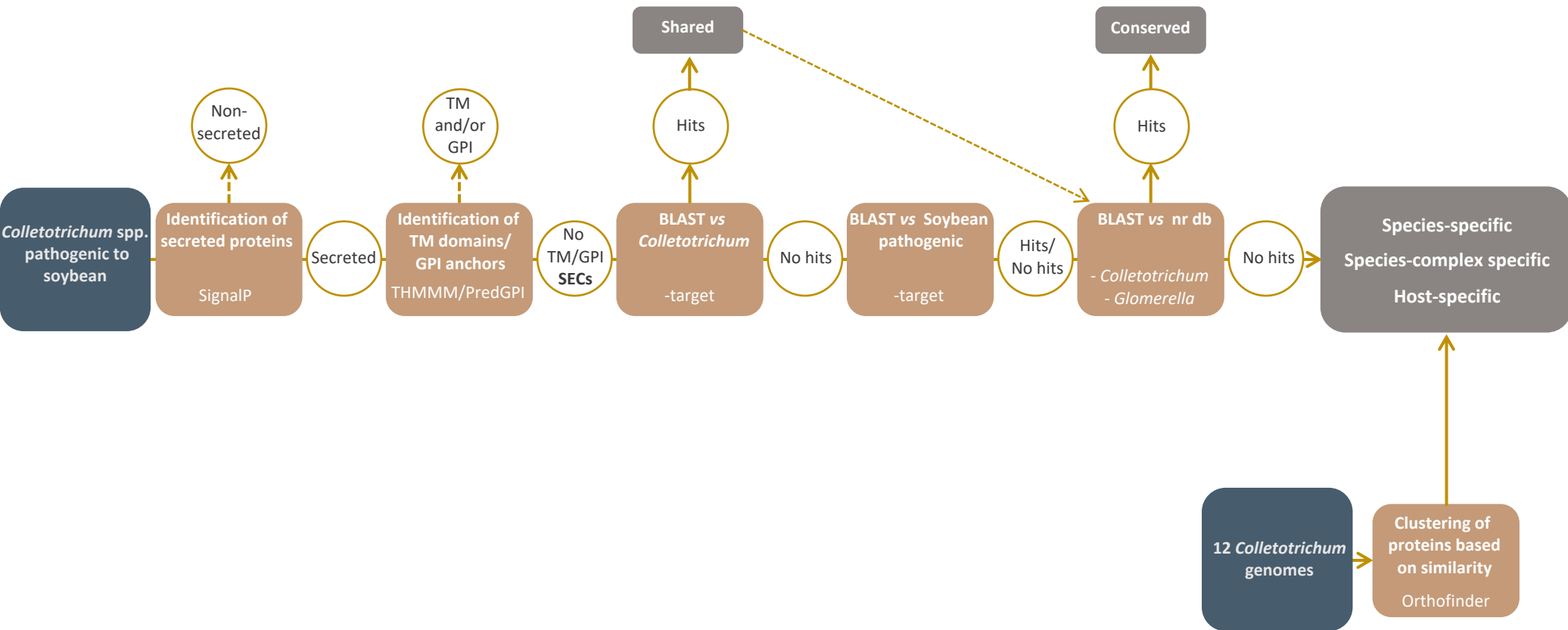

Supplement: Supplementary file 1 [file pathogens-10-01520-s001.zip › figure_S1.pdf]
